# Isolation of Female Germline Stem Cells from Mouse and Human Ovaries by Differential Adhesion

**DOI:** 10.1155/2022/5224659

**Published:** 2022-09-07

**Authors:** Maryam Saber, Pouya Tavakol, Fereshteh Esfandiari

**Affiliations:** Department of Stem Cells and Developmental Biology, Cell Science Research Center, Royan Institute for Stem Cell Biology and Technology, ACECR, Tehran, Iran

## Abstract

Spermatogonial stem cell (SSC) counterparts known as female germline stem cells (fGSCs) were found in the mammalian ovary in 2004. Although the existence of fGSCs in the mammalian postnatal ovary is still under controversy, fGSC discovery encourages investigators to better understand the various aspects of these cells. However, their existence is not accepted by all scientists in the field because isolation of fGSCs by fluorescent activated cell sorting (FACS) has not been reproducible. In this study, we used differential adhesion to isolate and enrich fGSCs from mouse and human ovaries and subsequently cultured them *in vitro*. fGSCs were able to proliferate *in vitro* and expressed germ cell-specific markers Vasa, Dazl, Blimp1, Fragilis, Stella, and Oct4, at the protein level. Moreover, mouse and human fGSCs were, respectively, cultured for more than four months and one month in culture. Both mouse and human fGSCs maintained the expression of germ cell-specific markers over these times. *In vitro* cultured fGSCs spontaneously produced oocyte-like cells (OLCs) which expressed oocyte-relevant markers. Our results demonstrated that differential adhesion allows reproducible isolation of fGSCs that are able to proliferate *in vitro* over time. This source of fGSCs can serve as a suitable material for studying mechanisms underlying female germ cell development and function.

## 1. Introduction

Long-standing dogma that has claimed a fixed number of follicles existing in the mammalian ovary has been challenged recently as neo-oogenesis has been observed in the adult mouse ovary [1]. Subsequent isolation and culture of female germline stem cells (fGSCs) from mouse and human ovaries by fluorescent activated cell sorting (FACS) and magnetic activated cell sorting (MACs) reinforced the postnatal oogenesis [2, 3]. In these studies, fGSCs were sorted using the germ cell marker DDX4. However, sorting live fGSCs by application of this antibody is controversial due to its cytoplasmic domain [4]. The FACS-based isolation method yields a few number of mitotically active germline cells (<0.1% cells per ovary) [5]. While the existence of fGSCs is the subject of extensive debate [4], their male counterparts (spermatogonial stem cells; SSCs) have been well characterized for many years [6]. SSCs are isolated by a simple and common method based on the differential attachment potential of germ cells and somatic cells [6]. This method enriches the SSC population simply by preparation of a gelatin-coated plate for trapping somatic cells and removes them from culture [7, 8]. This feasible approach was recently used for the enrichment of mouse fGSCs [9]. However, it remains to be answered whether differential adhesion allows the isolation of fGSCs from human ovaries. In this study, we intended to enrich fGSCs from human and mouse adult ovaries by the differential adhesion method. Easy culture of fGSCs could provide an *in vitro* source of material for studying mechanisms underlying female germ cell development and function. Moreover, it may serve as a suitable source for female infertility treatments in future reproductive medicine.

## 2. Materials and Methods

### 2.1. Animal

In this study, we used female NMRI mice (6-8 weeks old, from Pasteur Institute, Tehran, Iran). The mice were housed under a standard animal facility (controlled atmosphere with 12 : 12 h light/dark cycles and temperature of 20–25°C). They had free access to water and food. All animal care studies and procedures were approved by the Royan Institutional Review Board and Institutional Ethical Committee of Royan Institute (No.: Ec/92/1026).

### 2.2. Mouse Ovarian Cell Isolation

For the preparation of cells, ovaries from four mice were pooled and isolated using a two-step enzymatic digestion method involving a 15 min incubation in the enzymatic solution containing collagenase type IV (800 U/ml prepared in HBSS) and DNase-I (1 *μ*g/ml, Sigma-Aldrich) and a 10 min incubation with 0.05% trypsin-EDTA. When most of the cells were dispersed, the ovarian cells were washed 2 to 4 times in HBSS and centrifuged at 300 g for 5 min. Next, the supernatant was carefully removed from the pellet, the pellet was resuspended, and clumps of cells were removed by passing the suspension through a 70 *μ*m nylon. Finally, the supernatant was removed, and the cells were resuspended in culture medium. Isolated cells were plated onto gelatin-coated culture plates (3 × 10^5^ cells/per 3 cm^2^ plate, Falcon) in the culture medium containing *α*-MEM (Invitrogen) supplemented with 10% Fetal Bovine Serum (FBS) (Hyclone), 1 mM sodium pyruvate (Invitrogen), 1 mM nonessential amino acids (Invitrogen), 1x penicillin-streptomycin-glutamine (Invitrogen), 0.1 mM *β*-mercaptoethanol (Sigma-Aldrich), 1x concentrated N-2 supplement (R&D), 10^3^ units/ml leukemia inhibitory factor (LIF, Royan Institute), 10 ng/ml recombinant human epidermal growth factor (rhEGF, Royan BioTech), 1 ng/ml basic fibroblast growth factor (bFGF, Royan BioTech), and 40 ng/ml glial cell-derived neurotropic factor (GDNF, Royan BioTech). After 30 to 60 min of culture at 37°C, the supernatant that contained cells unattached to the plate was collected and transferred to a new 24-well plate coated with feeder cells (mitomycin C-treated MEFs). The medium was changed every 2–3 days, and when the cells reached confluence, they were digested using 0.05% trypsin (Invitrogen) followed by neutralization by adding 10% FBS and replated on fresh MEF at a 1 : 2 split ratio. The cells were cultured for four months.

### 2.3. Human Sample

Ovarian biopsies were collected from women (*n* = 6) with a mean age of 30 years (20-40 years), who underwent total abdominal hysterectomy for uterine fibroma or ovariectomy due to various gynecological pathologies other than ovarian pathology, infection, malignancy, or other pathologies. The study was performed after obtaining written informed consent from patients to use ovarian samples for research purposes. The tissue biopsies were collected during surgeries at Atieh Hospital and transferred in *α*-MEM media containing antibiotics (penicillin 100 U/ml and streptomycin 100 mg/ml; Invitrogen) on ice, to Royan Institute as soon as possible. The Royan Institutional Review Board and Institutional Ethical Committee of Royan Institute (No.: Ec/92/1026) approved the use and preparation of the human ovarian samples in this study.

### 2.4. Human Ovarian Cell Isolation

Ovarian biopsies were first washed several times in HBSS that contained antibiotics and then cut into small pieces by using a scalpel and incubated in enzyme solution. Enzymatic digestion was performed based on the mouse model. The human fGSCs were cultured and maintained for more than one month. However, after passaging, cells did not develop or grow further.

### 2.5. Viability Assessment

After isolation of human and mouse tissue, at least 100,000 cells from the whole ovarian cells were harvested and aliquoted into FACS tubes. Then, the cells were washed two times with PBS and centrifuged at 300 g for 5 minutes. Then, the buffer was decanted from the pelleted cells, and the cells were resuspended in 100 *μ*l of flow cytometry staining buffer; then, 5-10 *μ*l of propidium iodide (PI) staining solution was added to each sample, mixed gently, and incubated for 1 minute in the dark. Finally, PI fluorescence was determined by a BD FACS Aria II instrument.

### 2.6. Immunofluorescence Staining

Cultured ovarian cells were washed with PBS and fixed in 4% PFA for 20 min. After permeabilization by 0.5% Triton X-100, the cells were incubated for 1 h in blocking buffer that consisted of PBS and 10% normal goat serum followed by an overnight incubation period with primary antibody (Table S1) in a humidified chamber at 4°C. The cells were subsequently incubated with an appropriate secondary antibody at room temperature for 45 min. For nucleus staining, 4,6-diamidino-2-phenylindole (DAPI; Sigma-Aldrich) was used. Immunostaining without primary antibodies was used for negative control cells.

## 3. Results

### 3.1. Isolation of Female Germline Stem Cells from Mouse Ovaries by Differential Adhesion

**Figure 1:**
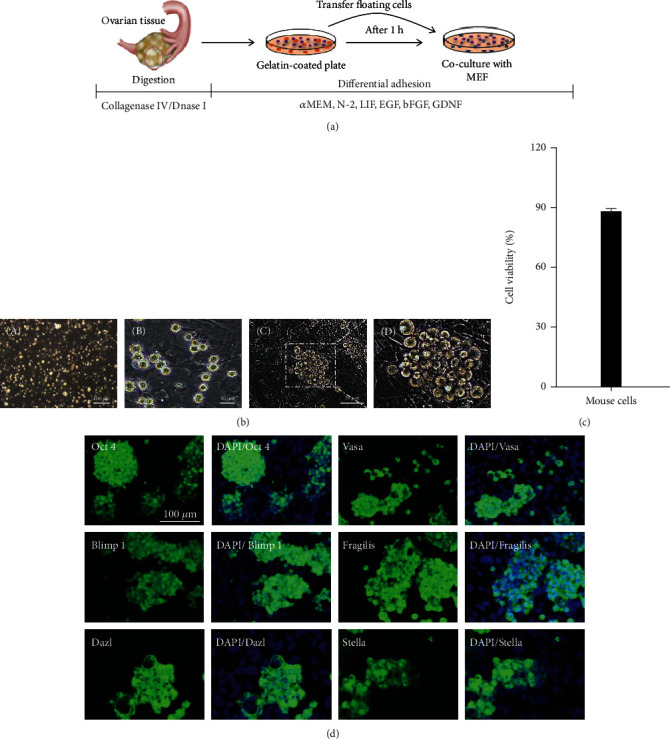
Isolation and culture of mouse female germline stem cells. (a) Schematic illustration of the culture system used for fGSC expansion. (b) Phase contrast images of mouse ovarian cell suspension after dissociation (A) and after 5 days of culture (B); spherical or grape-like clusters on day 30 in culture on MEF-coated plates (C, D; D at high magnification). (c) Viability percentage of mouse ovarian cells after ovarian tissue isolation by using PI staining analyzed by a BD FACS Aria II. (d) Characterization of established fGSCs. Expression of germ cell-specific markers in fGSCs detected by immunostaining analysis. The feeder cells were negative for these markers.

The procedure for isolation of fGSCs is schematically presented in Figure 1(a). Cell suspension achieved from the mouse ovary using enzymatic digestion (Figure 1(b), A) showed >80% viability (Figure 1(c)). Five days after transferring the supernatant cells to the MEF-coated plates, we observed small round cells (about 5-10 *μ*m) with little cytoplasm and a large ratio of nuclear plasma (Figure 1(b), B) similar to the FACS-sorted Ddx4-positive oogonial stem cells reported earlier [2]. The number of these cells increased during the culture, and they formed spherical or grape-like clusters without smooth borders (Figure 1(b), C and D).

### 3.2. Characterization of Mouse Primary Female Germline Stem Cells

In order to characterize fGSCs, we carried out immunofluorescence analysis. Immunostaining showed that the cells were strongly positive for germline markers (Blimp1, Stella, Fragilis, Vasa, Dazl, and Oct-4 (Figure 1(d)) on day 20 of culture. These markers were not detected in mitotically inactive mouse embryonic fibroblasts (MEFs) used as a feeder (Figure 1(d)).

### 3.3. Propagation of Mouse Female Germline Stem Cells in Culture by Differential Adhesion

**Figure 2:**
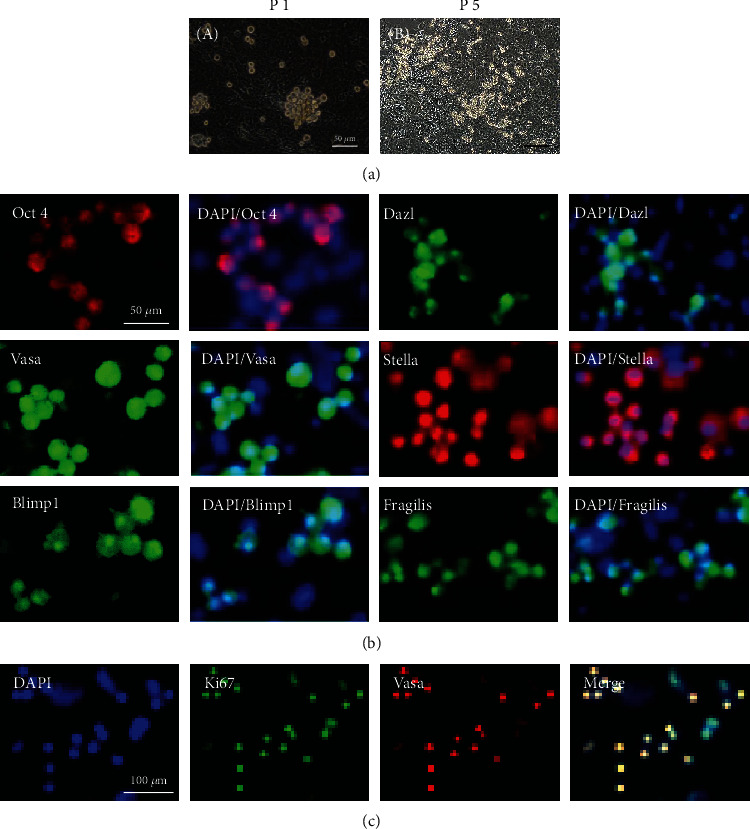
Characterization of established female mouse germline stem cells. (a) Phase contrast microscopy of cultured cells during passaging (day 30 at different passages (A: after passage 1; B: after passage 5) of fGSCs on MEF-coated plates. (b) Immunofluorescence staining for germline markers (Oct4, Vasa, Dazl, Blimp1, Stella, and Fragilis) in fGSCs (after 2 passages) in MEF-coated plates. Cells were counterstained with 4,6-diamidino-2-phenylindole (DAPI, blue) to visualize the nucleus. (c) Assessment of GSC proliferation by dual immunofluorescence for Vasa (red) expression and Ki67 incorporation (green).

Mouse fGSCs proliferated during the culture and were passaged every 10-20 days. The cells were passaged for four months (Figure 2(a)). Immunostaining revealed expression of germ cell-specific markers in fGSCs after long-term culture (≥4 months) (Figure 2(b)). Moreover, dual staining for Vasa and Ki67 (as a proliferative marker) demonstrated that fGSCs are actively proliferative in the culture (Figure 2(c)).

### 3.4. *In Vitro* Differentiation of Mouse Female Germline Stem Cells to Oocyte-Like Cells

**Figure 3:**
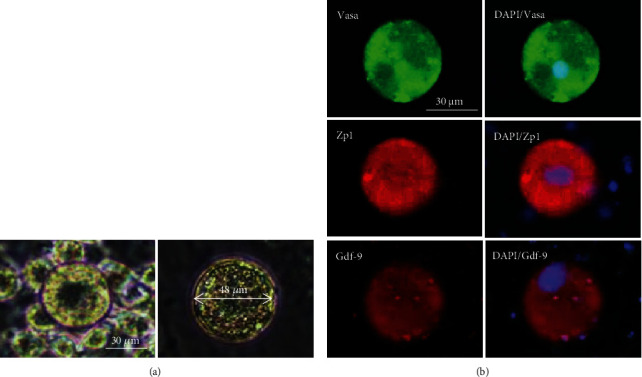
Characteristics of oocyte-like cells generated from female germline stem cells (fGSCs). (a) Phase contrast images of OLCs. Spontaneous generation of large spherical cells that morphologically resembled oocytes. (b) Immunofluorescence detection of germ cell and oocyte-specific markers in OLCs.

During *in vitro* culture of mouse fGSCs, they spontaneously differentiated into large spherical cells (diameter up to 48 *μ*m) and were morphologically similar to oocyte ((oocyte-like cells (OLCs)) (Figure 3(a)). We examined the expression of oocyte-specific markers in OLCs. Immunofluorescence analysis showed the expression of Gdf9, Vasa, and Zp1 (which are specific to the oocyte stage) at the protein level in OLCs (Figure 3(b)).

### 3.5. Isolation of Female Germline Stem Cells by Differential Adhesion from Human Ovaries

**Figure 4:**
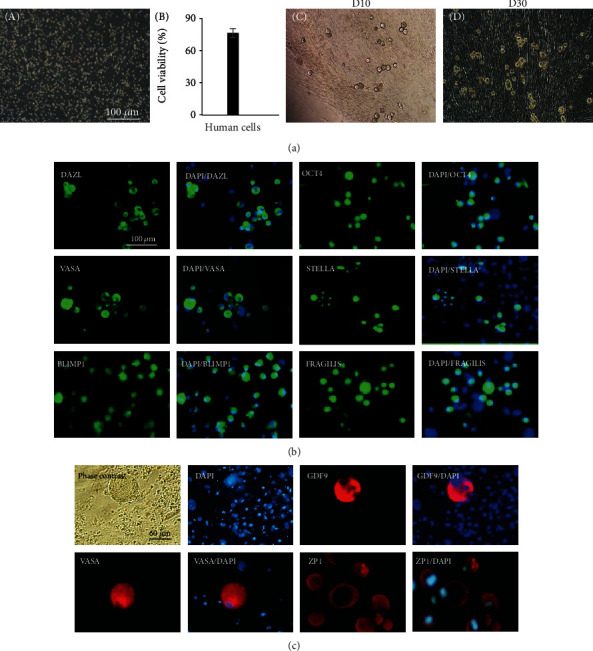
Characteristics of human female germline stem cells. (a) Phase contrast images of human ovarian cells after the isolation (A) and culture on different days (C and D); viability percentage of human ovarian cells after the isolation by using PI staining analyzed by BD FACS Aria II. (b) Immunofluorescence staining for germline markers in fGSCs on day 20 of culture in MEF-coated plates. (c) OLCs formed spontaneously from the fGSCs in the culture. Phase contrast and immunofluorescence staining images for oocyte markers (GDF9, ZP1, and VAZA) of differentiated OLCs in the culture.

Ovarian cells isolated from human ovarian biopsies showed >70% viability. fGSCs were morphologically detectable among other cells as round cells with a large nucleus to cytoplasm ratio, and they were undergoing mitosis in culture (Figure 4(a)). It was also possible to maintain the human fGSCs in culture for more than one month. We have been successful to isolate fGSCs from all 6 human samples. However, after passage, cells did not develop or grow further. Human fGSCs showed positive immunofluorescence staining for specific germline markers (BLIMP1, STELLA, FRAGILIS, VASA, DAZL, and OCT-4) on day 20 of culture. These markers were not detected in mitotically inactive mouse embryonic fibroblasts (MEFs) used as a feeder (Figure 4(b)). During the culture, the cells with a diameter of approximately 60 *μ*m (oocyte-like cells) developed among proliferating small cells. These cells were positive for germ and oocyte markers including VASA, GDF9, and VASA in immunofluorescence staining (Figure 4(c)).

## 4. Discussion

In the present study, we showed that differential adhesion allows efficient isolation and subsequent culture of fGSCs from both mouse and human ovaries. This approach allowed easy enrichment and culture of fGSCs that expressed germ cell markers in culture. Among different methods of cell isolation, the differential adhesion method is fast, easy, of low cost, and safe; this method causes the least negative effects on cell viability [10] [11] and is commonly used for *in vitro* cultures of male germ cells [12–14]. As long as 48 years ago, Steinberger and Steinberger [8] described that germ cells are enriched in the supernatant while somatic cells preferentially attach to the culture dish in single-cell suspensions of rats. A preferential expression of germ cell markers in the supernatant was also seen by Eildermann et al. [12] for marmoset testicular cell cultures and by Sadri-Ardekani et al. [14] and Kossack et al. [13] for human testicular cell cultures. It was proven that fibroblasts (MEFs) as the most commonly used feeder cells act as microniche by allowing secure attachment and proliferation of germ cells and secrete certain components into the media that support germ cell proliferation [15].

Compared to the FACS method for fGSC isolation, differential adhesion shortens the time of enrichment and provides the condition for accelerating the fGSC proliferation *in vitro*. In our experience, FACS-isolated fGSCs reached the confluency after 30 days of isolation (data not shown) while those isolated by differential adhesion reached the confluency 20 days after plating. It may be related to the few number of cells that are isolated by FACS.

We were able to culture the mouse fGSCs for three months in this system, and these cells maintained the expression of germ cell-specific markers (Vasa, Dazl, Blimp1, Stella, Fragilis, and Oct4) during this time. Moreover, fGSCs that were cultured by differential adhesion increased in number and efficiently expressed a proliferation marker (Ki67) demonstrating active proliferation of the cells. We also found that during *in vitro* culture, fGSCs spontaneously produced OLCs with morphology and gene expression profile similar to native oocytes. The maximum size that OLCs grew was, respectively, 48 and 60 *μ*m in mouse and human, and they did not show strong expression of Sycp3 (data not shown) which is expressed during meiosis [16] that showed that they were at early stages of development. Improving culture conditions may promote OLC development *in vitro*. We used a culture medium which has been reported [3] previously to be suitable for the propagation of fGSCs. One of the most important factors in this media is LIF which promotes fGSC proliferation and inhibits differentiation [17, 18]. However, we could observe a differentiation leakage in this condition. The oocyte differentiation was very low in this condition, about 1% which is ignorable. It was shown that medium supplementation with follicle-stimulating hormone (FSH) and luteinizing hormone (LH) or the presence of cumulus cells in *in vitro* culture condition [19] leads to *in vitro* maturation of oocyte [20–22]. So, in order to study the differentiation potential of fGSCs, we used a different culture media supplemented with 10% FBS, 10 ng/ml hEGF, 5 *μ*l/ml insulin/transferrin/selenium, 0.05 IU follicle-stimulating hormone (FSH), and 0.03 IU luteinizing hormone (LH). Moreover, we removed LIF from fGSCs which is another inducer for oocyte differentiation [23, 24]. When using this differentiation media, more oocytes were differentiated, and the size of oocytes which was differentiated in this condition was larger than those formed spontaneously during propagation (the data have not been reported). Further investigations are required to reach optimal conditions for achieving more developed OLCs.

In line with our study, the isolation of fGSCs from mouse ovaries was reported recently [9]. However, they did not apply this method to the isolation of fGSCs from human ovaries. Here, we showed efficient isolation of fGSCs from human ovaries by differential adhesion for the first time. Moreover, ovarian surface scarping has been used for the isolation of fGSCs in humans. This method was employed for the first time in 2004 by the Bukovsky group [25] and then by Virant-Klun et al. [26] and Parte et al. [27] in order to isolate fGSCs from the ovarian surface epithelium (OSE) of the human ovaries. The initial number of cells that were obtained by using these methods is very low, and the scraped cells (putative fGSCs) underwent spontaneous differentiation not only into the OLCs but also into other cells such as neural-like cells [16].

## 5. Conclusion

In summary, we established the differential adhesion method for fGSC isolation from mouse and human ovaries. The fGSCs expressed germ cell-specific markers in culture and proliferated actively over time. Importantly, they were able to differentiate into OLCs *in vitro*. This culture system allows enrichment of fGSCs to serve as an *in vitro* model for studying basic aspects of reproductive biology and as a promising source to be used in future reproductive medicine.

## Data Availability

All data generated or analyzed during this study are included in this article. Further enquiries can be directed to the corresponding author.

## Abbreviations

SSCs: Spermatogonial stem cells
fGSCs: Female germline stem cells
FACS: Fluorescent activated cell sorting
MACs: Magnetic activated cell sorting
OLCs: Oocyte-like cells
MEFs: Mouse embryonic fibroblasts.

## Supplementary Materials

Supplementary Materials The list of antibodies used for immunofluorescence staining has been illustrated as a table in the supplementary file.
