# Supplementary material for: Isolation of Female Germline Stem Cells from Mouse and Human Ovaries by Differential Adhesion
Source: Int J Cell Biol. 2022 Sep 7;2022:5224659. doi: 10.1155/2022/5224659 (PMC9473869; doi:10.1155/2022/5224659)
Supplement: Supplementary Materials — The list of antibodies used for immunofluorescence staining has been illustrated as a table in the supplementary file. [file 5224659.f1.docx]

**Supplementary table**

**Table S1. Antibodies used for immunofluorescence staining.**

| **Antibody** | **Company** | **Catalog number** | **Concentration** |
| --- | --- | --- | --- |
| **Oct4** | Abcam | Ab19857 | 1:100 |
| **Vasa** | Abcam | Ab13840 | 1:100 |
| **Stella** | Abcam | Ab19878 | 1:100 |
| **STELLA** | Santa Cruz | Sc376862 | 1:100 |
| **Blimp1** | Abcam | Ab81961 | 1:50 |
| **Dazl** | Abcam | Ab34139 | 1:100 |
| **Fragilis** | Abcam | Ab15592 | 1:100 |
| **Gdf9** | Santa Cruz | Sc7407 | 1:100 |
| **Ki67** | Abcam | Ab15580 | 1:100 |
| **Zp1** | Santa Cruz | Sc23706 | 1:100 |
| **Goat anti-mouse** | Invitrogen | A28175 | 1:500 |
| **Goat anti-mouse** | Invitrogen | A11005 | 1:500 |
| **Goat anti-rabbit** | Abcam | Ab96899 | 1:500 |
| **Goat anti-rabbit** | Invitrogen | A11072 | 1:500 |
| **Donkey anti-goat** | Invitrogen | A11057 | 1:500 |
